# Poly-**γ**-Glutamic Acid Attenuates Angiogenesis and Inflammation in Experimental Colitis

**DOI:** 10.1155/2013/982383

**Published:** 2013-05-16

**Authors:** Munkhtugs Davaatseren, Jin-Taek Hwang, Jae Ho Park, Myung-Sunny Kim, Shuaiyu Wang, Mi Jeong Sung

**Affiliations:** ^1^Research Division Emerging Innovative Technology, Korea Food Research Institute, 516 Baekhyun-Dong, Bundang-Ku, Seongnam Gyeonggi 463-746, Republic of Korea; ^2^University of Science & Technology, Daejeon 305-350, Republic of Korea; ^3^Yellow Sea Fisheries Research Institute, Chinese Academy of Fishery Sciences, 106 Nanjing Road Qingdao, Shandong 266071, China

## Abstract

Poly-**γ**-glutamic acid (**γ**-PGA), naturally secreted from various strains of *Bacillus*, has anti-inflammatory activity. In inflammatory bowel disease (IBD), inflammation is promoted and sustained by angiogenesis; however, the role played by **γ**-PGA in this condition is unclear. Therefore, we evaluated **γ**-PGA effects on angiogenesis and inflammation in a dextran sulfate sodium- (DSS-) induced mouse colitis model. Experimental colitis was induced in male C57BL/6 mice by administering 3% DSS. Disease activity index (DAI), histopathological scores, microvascular density, myeloperoxidase activity, and VEGF-A and VEGFR2 expression were compared among control mice, DSS-treated mice, and mice receiving 3% DSS along with **γ**-PGA at 50 mg/kg body weight per day or 3% DSS with **γ**-PGA at 200 mg/kg body weight per day. We found that **γ**-PGA significantly attenuated weight loss, DAI, and colon shortening. **γ**-PGA also significantly reduced histopathological evidence of injury. Moreover, **γ**-PGA significantly attenuated DSS-induced blood vessel densities. Furthermore, **γ**-PGA attenuated DSS-induced expression of VEGF-A and its receptor, VEGFR2. In addition, **γ**-PGA treatment led to reduced recruitment of leukocytes to the inflamed colon. Therefore, our results indicate that **γ**-PGA has potential application in conditions marked by inflammatory-driven angiogenesis and mucosal inflammation.

## 1. Introduction

Inflammatory bowel disease (IBD) is characterized by chronic inflammation and is associated with extensive tissue injury and remodeling caused by tissue edema, inflammatory cell infiltration, loss of epithelial integrity, and increased angiogenesis [1, 2]. These features contribute to IBD pathophysiology, involving several cell types and mediators, through diverse mechanisms. 

Angiogenesis, formation of new capillaries from preexisting vessels is intimately involved in a number of biological processes, including growth, development, and repair [3, 4]. In the development of human and experimental IBD, angiogenesis contributes to the remodeling of blood vessels, in which vascular endothelium is responsible for the recruitment of leukocytes to the site of inflammation via upregulation of adhesion molecules and secretion of chemokines [5, 6]. In turn, endothelial cells respond to the proangiogenic cytokines produced by the infiltrating cells through a process referred to as immune-driven angiogenesis [7, 8]. 

The angiogenic role played by pathways involving vascular endothelial growth factor-A (VEGF-A) and its receptors is well characterized. VEGF-A is known to be a fundamental mediator of pathologic angiogenesis in several inflammatory disorders, such as neoplasia and chronic inflammation, including IBD [9, 10]. VEGF-A triggers endothelial proliferation and also causes vascular leakage and leukocyte infiltration [11]. Evidence of VEGF in IBD upregulation in response to inflammatory signaling via TNF*α* further supports the existence of a direct link between inflammation and angiogenesis [12, 13]. Therefore, decreased VEGF levels should lead to decreased angiogenesis and inflammation in human and experimental IBD.

Poly-*γ*-glutamic acid (*γ*-PGA) is naturally secreted from various strains of *Bacillus* during the process of soybean fermentation and is a safe and edible polymer in which the *α*-amino and *γ*-carboxylic acid groups of D- or L-glutamic acid are linked by isopeptide bonds [14]. *γ*-PGA is water soluble, biodegradable, edible, and nontoxic [15]. Furthermore, recent reports have indicated immune modulatory activities of *γ*-PGA or its derivatives. For instance, oral administration of high molecular mass *γ*-PGA (2000 kDa) enhanced NK cell-mediated antitumor activity in mice bearing MHC class I-deficient tumors [16]. Nanoparticles, composed of *γ*-PGA and L-phenylalanine ethylester, served as an adjuvant, which was able to activate dendritic cells (DC) and enhance adaptive immune responses against antigens carried by the nanoparticles [17]. *Bacillus*-derived *γ*-PGA regulates the developmental pathways of naive CD4 T cells through antigen-presenting cell-dependent and -independent mechanisms [18]. Moreover, recently, Lee et al. reported that *Bacillus*-derived *γ*-PGA attenuates allergic airway inflammation through a toll-like receptor-4-dependent pathway in a murine model of asthma [19]. These findings led us to examine the protective effects of *γ*-PGA on intestinal inflammation. While angiogenesis has been shown to promote and sustain many events in inflammation, the role that *γ*-PGA plays in the modulation of intestinal inflammation is less clear. Therefore, in this study, we evaluated the effects of *γ*-PGA on angiogenesis and inflammation in a DSS-induced mouse colitis model. 

Here, we showed that *γ*-PGA significantly attenuated weight loss, the disease activity index (DAI), and colon shortening in an animal model of colitis. *γ*-PGA significantly reduced histopathological evidence of injury; moreover, blood vessel densities were significantly elevated in DSS-treated mice, but the elevation was significantly attenuated in *γ*-PGA mice. Furthermore, *γ*-PGA attenuated DSS-induced expression of VEGF-A and its receptor VEGFR2. In addition, *γ*-PGA reduced recruitment of leukocytes to the inflamed colon. Our results suggest beneficial effects of *γ*-PGA in conditions marked by inflammatory-driven angiogenesis and mucosal inflammation.

## 2. Materials and Methods

### 2.1. Induction of Experimental Colitis

Mice used in this study were 8-week-old male C57BL/6 mice (Charles River Korea, Seoul, Republic of Korea), which were housed in the Korea Food Research Institute (KFRI) at a temperature of 22–26°C, under a 12 h/12 h light/dark cycle, with free access to water. Mice were allowed to adapt to their food and environment for 1 week before the start of the experiment. 

The animals were matched on body weight and then randomly assigned into the following 4 groups of 8 mice each: control group (without DSS; control), 3% dextran sulfate sodium (DSS) administration group (DSS), 3% DSS with *γ*-PGA at 50 mg/kg body weight per day (D + P50), and 3% DSS with *γ*-PGA at 200 mg/kg body weight per day (D + P200). Experimental colitis was induced in mice by supplementing drinking water with filter-purified 3% DSS [20, 21], to which they had free access (DSS, MW 1/4 36–50 kDa; MP Biochemicals, Aurora, OH, USA); mice in the control group received tap water without DSS, for 7 days. *γ*-PGA (50 or 200 mg/kg body weight, dissolved in water) or vehicle (water) was administered orally for 7 days, commencing at the same time as exposure to DSS. 

Administration of 3% DSS produces an erosive distal colitis, with an initial onset at 3 or 4 days, and is characterized by progressive weight loss, diarrhea, occult blood, leukocyte infiltration, colon shortening, loss of intestinal epithelial barrier, and histopathological changes in colon structure. Mice typically lose approximately 20% of their body weight by day 7 when exposed to continuous administration of 3% DSS. Thus, mice were assessed daily for colitis through monitoring of body weight, stool form, occult blood, and survival [22]. The DAI was determined by averaging scores of weight loss, stool form, and occult blood. Scores were defined by (1) change in weight (0: <1%; 1: 1–5%; 2: 5–10%; 3: 10–15%, 4: >15%); (2) occult blood (0: negative, 2: positive, 4: gross bleeding); and (3) stool form (0: normal; 2: loose stools; 4: diarrhea), as previously described [23–25]. 

On day 7, mice were sacrificed by cardiac puncture (under ketamine/xylazine anesthesia). Histological samples were fixed in cold 4% phosphate-buffered formalin or frozen at −20°C for myeloperoxidase (MPO) and western blotting analysis.

### 2.2. Histopathological Analysis

Formalin-fixed colon sections were paraffin-embedded and 10 *μ*m sections were stained with hematoxylin and eosin (H&E). The degree of colitis was scored by an examiner without prior knowledge of experimental procedures, as described previously [26]. Assessment included noting of edema, extent of injury, leukocyte infiltration, crypt abscesses, and loss of goblet cells [27]. In this grading system, inflammation severity was scored on a 0–3 scale, (0: none; 1: slight; 2: moderate; 3: severe), as was the extent of injury (0: none; 1: mucosal; 2: mucosal and submucosal; 3: transmural); crypt damage was scored on a 0–4 scale (0: none; 1: basal third damaged; 2: basal two-thirds damaged; 3: only surface epithelium intact; 4: loss of entire crypt and epithelium). Each value was multiplied by an extent index ranging from 1 to 4 that reflects the amount of involvement for each section (1: 0–25%, 2: 26–50%, 3: 51–75%, 4: 76–100%). At least 3 sections from each colon were analyzed to produce each score value. The scoring system yields minimal and maximal total scores of 0 and 40, respectively.

### 2.3. Immunofluorescence Analysis

Frozen colon tissues were processed for immunofluorescence analysis using a hamster anti-mouse CD31 (Chemicon, Temecula, CA) and DAPI (Invitrogen, Carlsbad, CA) antibodies. The primary antibody was incubated on slides overnight at 4°C. Cy3-conjugated anti-hamster IgG antibody was used as secondary antibody. Immunofluorescence was visualized under a Nikon Eclipse T*i* confocal microscope (Thornwood, NY). Area densities (percentage of tissue area) of blood vessels in each colon were measured by noting CD31-immunopositive blood vessels, at a magnification of 200x in 5 regions, each amounting to a 0.21-mm^2^ area, per section. 

### 2.4. Western Blot Analysis

Immunoblotting was performed as described previously [23]. Each frozen colon tissue specimen (10–20 mg) was homogenized in phosphate-buffered saline (PBS) containing a protease inhibitor cocktail (Calbiochem, San Diego, CA), and the total protein concentration was quantitated. The samples (50 *μ*g of protein per lane) were mixed with sample buffer, boiled for 10 min, separated by SDS-polyacrylamide (10%) gel electrophoresis under denaturing conditions, and electroblotted onto nitrocellulose membranes. The membranes were incubated overnight at 4°C with anti-VEGF-A (Santa Cruz Biotechnology, Santa Cruz, CA, USA) and VEGFR-2 (GeneTex, San Antonio, TX) antibodies. The membranes were stripped and then reblotted with antiactin (dilution, 1 : 2,000; Sigma, St. Louis, MO) to verify equal protein loading in each lane. Experiments were repeated 3 times. 

### 2.5. Measurement of Tissue MPO Content

MPO activity was measured as previously described [28], with slight modifications. Samples (20–30 mg) of colon tissue were frozen in liquid nitrogen, crushed, and freeze-thawed 3x in 0.5% (w/v) hexadecyltrimethylammonium (HETAB, Sigma, St. Louis, MO) buffer. Samples were then sonicated for 10 s at 50% of maximum power and cleared by centrifugation at 10,000 ×g before measuring MPO activity in the supernatants using 0.1% *o*-dianisidine as substrate. The change in absorbance was read at 460 nm using a SpectraMax M2 Microplate Reader (Molecular Devices, Sunnyvale, CA). MPO activity was expressed as the amount of enzyme necessary to produce a change in absorbance of 1.0 unit per minute per gram of tissue (wet weight).

### 2.6. Statistical Analysis

All data are expressed as mean ± standard deviation (SD). Analysis of variance (ANOVA) was used for comparison between groups. A value of *P* < 0.05 was considered statistically significant.

## 3. Results

### 3.1. *γ*-PGA Reduces Body Weight Loss, DAI, and Shortening of the Colon in a DSS-Induced Colitis Model

**Figure 1:**
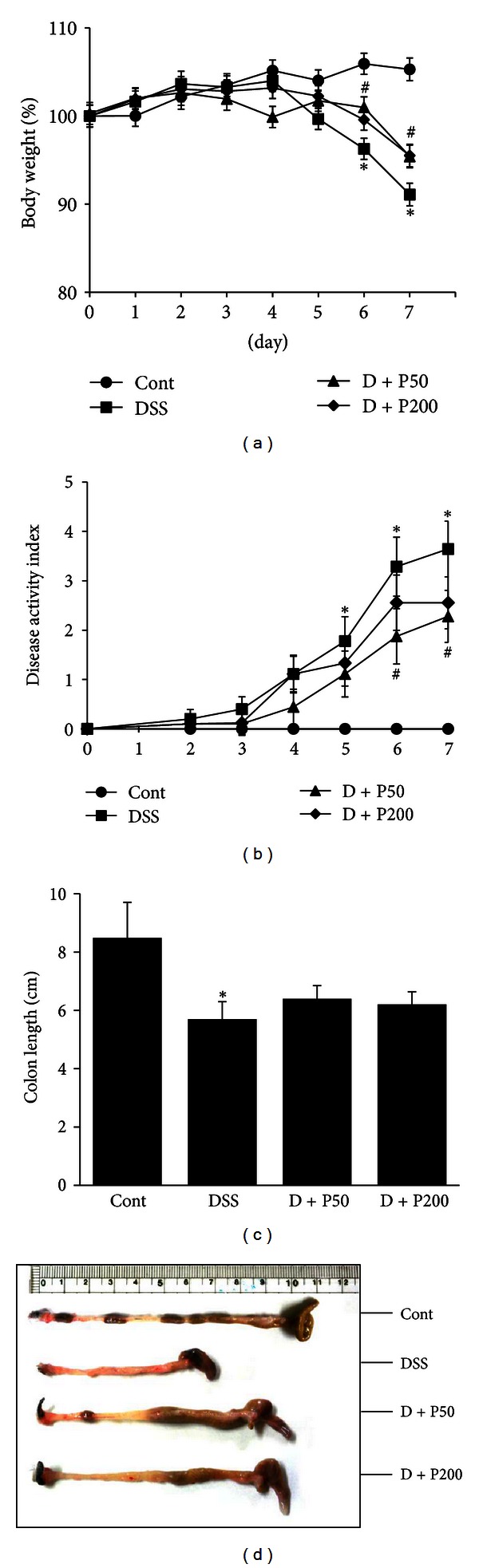
*γ*-PGA attenuates clinical signs in mice with DSS-induced colitis. (a) Changes in the body weights and (b) clinical scores of *γ*-PGA-treated mice and control mice administered 3% DSS were monitored every day. Body weight values are expressed as a percentage of body weight on day 0. (c) Colons were obtained from mice 7 days after commencement of DSS administration, and their lengths were measured. (d) Macroscopic features of the colons. Control group (without DSS; Cont), 3% DSS administration group (DSS), 3% DSS with *γ*-PGA at 50 mg/kg body weight per day (D + P50), 3% DSS with *γ*-PGA at 200 mg/kg body weight per day (D + P200). Data shown are from 3 independent experiments and are expressed as mean ± SD (*n* = 8 per group). *P < 0.05, DSS versus Cont; ^#^ *P* < 0.05, D + P50 and D + P200 versus DSS.

We first noted symptomatic parameters such as body weight loss, DAI, and shortening of the colon caused by colitis 7 days after the start of the 3% DSS oral challenge. The body weights of DSS-treated mice were markedly decreased compared to those of control mice. However, mice in the D + P50 and D + P200 groups showed less body weight loss than did DSS-treated mice (Figure 1(a)). The DAI, which measures common features of the DSS-induced model of colitis, also significantly decreased with *γ*-PGA treatment (Figure 1(b)). In addition, we found that the colon was significantly shorter in the DSS-treated mice than in the control mice. However, colon length in the D + PGA50 and D + PGA200 mice was increased as compared to that in DSS mice (Figures 1(c) and 1(d)). 

### 3.2. *γ*-PGA Reduced Histopathological Evidence in a DSS-Induced Colitis Model

**Figure 2:**
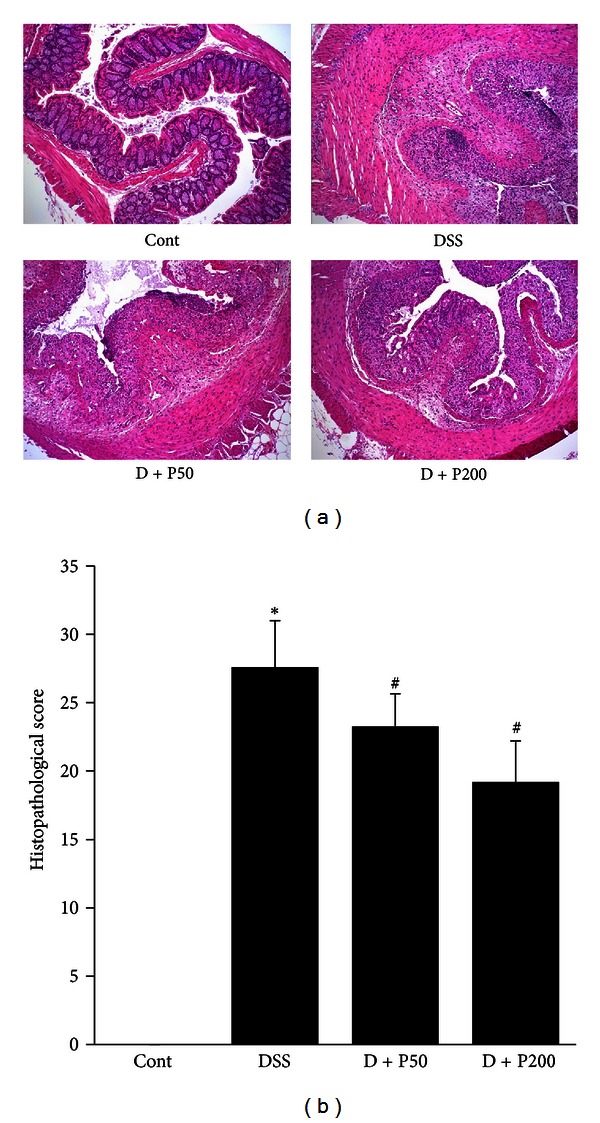
Histopathological examination in a **γ**-PGA-treated DSS-induced colitis model. (a) Colons were obtained 7 days after commencement of DSS administration and then sectioned and stained with hematoxylin and eosin. Control group (without DSS; Cont), 3% DSS administration group (DSS), 3% DSS with *γ*-PGA at 50 mg/kg body weight per day (D + P50), 3% DSS with *γ*-PGA at 200 mg/kg body weight per day (D + P200). Original magnification: 40x. (b) Histopathological scores of the analyzed slides. Bars represent the mean ± SD from 3 slides per mouse. *P < 0.05, DSS versus Cont; ^#^ *P* < 0.05, D + P50 and D + P200 versus DSS.

Histological examination revealed that DSS mice showed typical inflammatory changes in colonic architecture, such as ulceration, crypt dilation, and goblet cell depletion, as well as mixed cell infiltration, composed mainly of macrophages and lymphocytes [17]. However, histological analysis of the colons from D + P50 and D + P200 mice showed greatly reduced numbers of infiltrating cells, as well as a lesser degree of mucosal injury and edema (Figure 2(a)). In addition, histopathological examination of H&E-stained colonic sections under light microscopy was used to assess intestinal inflammatory status. Compared to control mice, DSS mice showed significantly increased histopathological scoring of disease. However, D + P50 and D + P200 mice showed a significant and dose-dependent decrease in inflammatory status (Figure 2(b)).

### 3.3. *γ*-PGA Reduced Angiogenesis in a DSS-Induced Colitis Model

**Figure 3:**
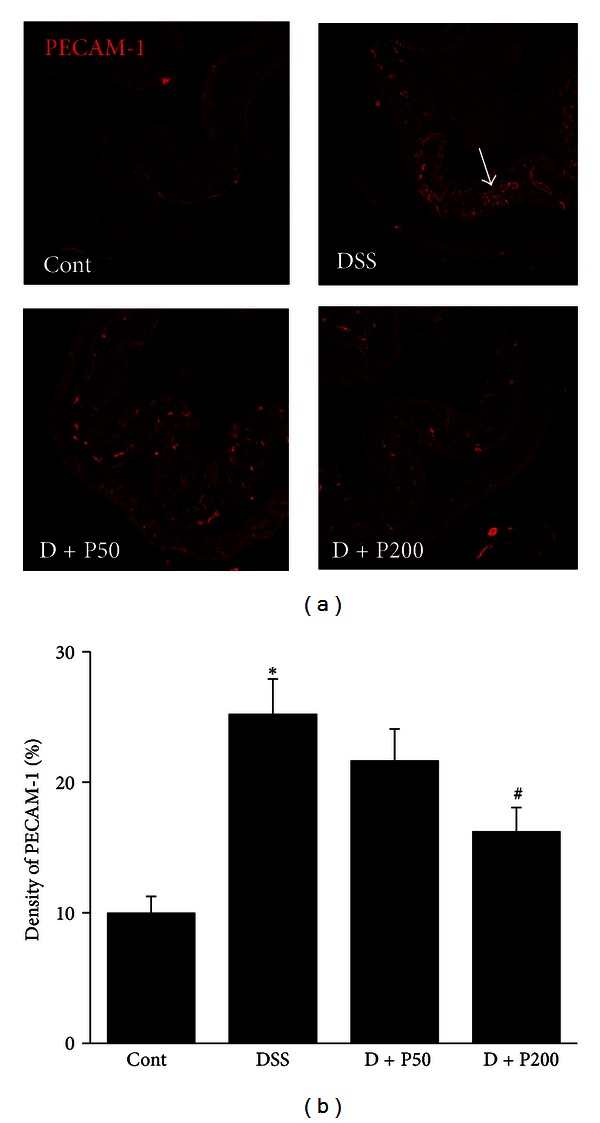
Angiogenesis in a **γ**-PGA-treated DSS-induced colitis model. (a) Colonic microvasculature stained for endothelial cells (200x magnification) using the CD31 antibody. Control group (without DSS; Cont), 3% DSS administration group (DSS), 3% DSS with *γ*-PGA at 50 mg/kg body weight per day (D + P50), 3% DSS with *γ*-PGA at 200 mg/kg body weight per day (D + P200). (b) Analysis of the area densities of blood vessels. Bars represent the mean ± SD from 3 slides per mouse. *P < 0.05, DSS versus Cont; ^#^ *P* < 0.05, D + P50 and D + P200 versus DSS.

To investigate the effects of PGA on angiogenesis in DSS-induced colitis, we measured the expression of CD31, a blood vessel endothelial cell marker, and quantified the microvascular density. Compared to control mice, DSS mice had a significantly increased microvascular density. Importantly, D + P50 and D + P200 mice had a significantly reduced number of mucosal vessels (Figure 3).

### 3.4. *γ*-PGA Reduced the Expression of VEGF-A and VEGFR2 in a DSS-Induced Colitis Model

**Figure 4:**
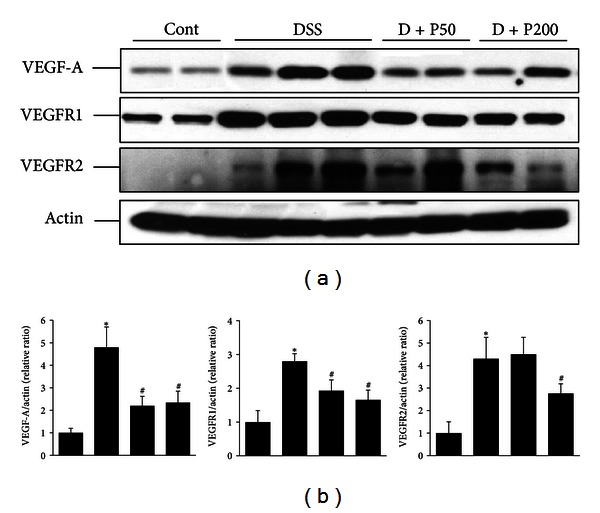
Expression of VEGF-A and VEGFR2 in a **γ**-PGA-treated DSS-induced colitis model. (a) VDGF-A and VEGFR2 expression levels were determined by immunoblotting in colon samples from the control group (without DSS; Cont), 3% DSS administration group (DSS), 3% DSS with *γ*-PGA at 50 mg/kg body weight per day (D + P50), 3% DSS with *γ*-PGA at 200 mg/kg body weight per day (D + P200). *β*-Actin was used as an internal control. (b) Densitometric analyses are presented as the relative ratio of each protein to actin. The ratio relative to the control is arbitrarily presented as 1. Bars represent the mean ± SD from 3 experiments. *P < 0.05 versus control; ^#^ *P* < 0.05 versus DSS.

VEGF-A is a well-characterized, fundamental mediator of pathologic angiogenesis [3]. To determine the effects of *γ*-PGA on the expression of VEGF-A and VEGFR-2 in DSS-induced colitis, we performed western blot analysis. Although VEGF-A was expressed in control mice, its expression was markedly enhanced in DSS mice. However, D + P50 and D + P200 mice demonstrated significantly decreased VEGF-A expression compared to that in DSS mice. Similarly, the increase in VEGFR2 was significantly reduced in D + P50 and D + P200 mice compared to DSS mice (Figure 4).

### 3.5. *γ*-PGA Reduced MPO Activity in a DSS-Induced Colitis Model

**Figure 5:**
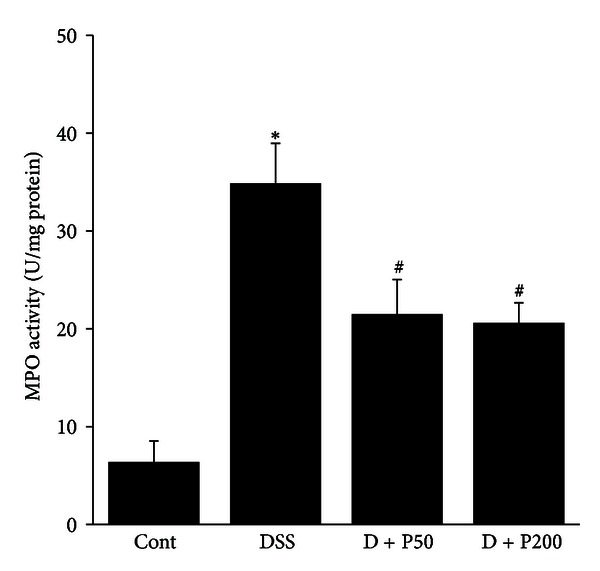
MPO activity in a **γ**-PGA-treated DSS-induced colitis model. Control group (without DSS; Cont), 3% DSS administration group (DSS), 3% DSS with *γ*-PGA at 50 mg/kg body weight per day (D + P50), 3% DSS with *γ*-PGA at 200 mg/kg body weight per day (D + P200). Data shown represent the results of 3 independent experiments, expressed as mean ± SD (*n* = 8 per group). *P < 0.05, DSS versus Cont; ^#^ *P* < 0.05, D + P50 and D + P200 versus DSS.

MPO is an enzyme produced mainly by polymorphonuclear leukocytes; its activity correlates with the degree of neutrophil infiltration in tissues. Therefore, to investigate the effects of *γ*-PGA on neutrophil infiltration in DSS-induced colitis, we measured MPO activity. MPO activity was markedly higher in DSS mice than in control mice. This increase in MPO activity was significantly reduced in D + P50 and D + P200 mice (Figure 5).

## 4. Discussion

It has now been clearly established that microvascular changes associated with angiogenesis are key components of the tissue injury and remodeling processes that necessarily accompany chronic inflammation [29, 30]. However, the important role played by angiogenesis in several chronic inflammatory diseases is still being elucidated [29]. Crohn's disease (CD) and ulcerative colitis (UC) are 2 major forms of IBD, an immune-mediated, chronic, relapsing intestinal inflammatory condition; although the process underlying development of colitis is quite complex, several animal models are routinely used to represent the pathophysiology of IBD. The DSS-induced colitis model has been widely used in research. Importantly, analysis of the DSS-induced colitis model revealed the involvement of a number of genes that play a role in angiogenesis. Therefore, we and other researchers have found this an acceptable model of colitis [31, 32].

DSS ingestion results in typical inflammatory changes in colonic architecture, such as ulceration, crypt dilation, goblet cell depletion, edema, as well as mixed cell infiltration, mainly involving neutrophils and macrophages [33]. In this present study, control mice demonstrated a normal epithelial surface with no evidence of inflammatory infiltrates or edema. By comparison, DSS-treated mice showed extensive interruptions of the epithelial surface, with submucosal edema and chronic inflammatory cell infiltration that consisted of dense lymphoid aggregates devoid of germinal centers. However, mice concomitantly given DSS and *γ*-PGA showed an attenuated tissue injury; samples from this group retained a relatively more intact epithelial surface, with little inflammation in the intestinal glands (Figure 2). Moreover, recently, several studies have shown that neutrophil infiltration, an important factor in inflammation, may be triggered by angiogenesis and that these events are causally associated [31, 34, 35]. Neutrophils that infiltrate the inflamed gut in IBD provide many of the cytokines, growth factors, proteolytic enzymes, and oxidants that are significant contributors to injury and inflammatory angiogenesis. These newly formed blood vessels supply the inflamed tissue with oxygen and nutrients, thereby allowing the transport of immune cells into the inflamed region [36–38]. In the present study, we found a highly significant reduction in neutrophil infiltration and histopathological evidence of inflammation, as well as reduced angiogenesis in mice treated with DSS and *γ*-PGA compared to mice receiving DSS alone (Figures 2, 3, and 5). These results suggest that *γ*-PGA plays a significant role in modulating the immune response involving the migration of leukocytes, especially neutrophils, into the inflamed tissue in IBD.

Angiogenesis is now accepted to contribute greatly to the inflammation observed in IBD; it also plays an important role in the repair of tissue and blood vessel injury [39]. Indeed, active microvessel growth has been noted in the acutely inflamed bowel tissues of patients with UC and CD, as well as in an experimental colitis model [31]. In this study, mice administered DSS showed markedly increased levels of the angiogenic marker CD31, whereas mice treated with *γ*-PGA demonstrated inhibition of angiogenesis (Figure 3). These results suggest that *γ*-PGA has an inhibitory effect on DSS-induced angiogenesis.

The angiogenic role played by the pathways involving VEGF isoforms and their receptors is well characterized. VEGF-A is the best characterized and is a fundamental mediator of pathogenic angiogenesis [40, 41]. Moreover, VEGF-A is key in several chronic inflammatory disorders, in which VEGF-A not only promotes pathologic angiogenesis, but also directly fosters inflammation. It is well known that VEGF-A is intimately involved in the pathogenesis of diseases such as rheumatoid arthritis, atherosclerosis, and chronic lung inflammation [42–44]. Recently, Scaldaferri et al. reported that blockade of VEGF-A reduced angiogenesis and inflammation in IBD patients and in a DSS-induced model of colitis [12]. In this study, we investigated the expression of VEGF-A and its receptors in the DSS-treated mice. We found that the mice showed markedly enhanced expression of VEGF-A and VEGFR2 compared to control mice; however, mice receiving concomitant DSS and *γ*-PGA showed attenuated VEGF-A and VEGFR2 expression. However, the expression of VEGFR1 was not different between control and DSS mice (data not shown). Similar to our data, Scaldaferri et al. reported that VEGF-A is upregulated in tissues involved in IBD in humans and in colitic mice, as is its receptor VEGFR-2; whereas VEGFR1 expression is not upregulated [12]. These results suggest that *γ*-PGA suppresses the DSS-induced VEGF pathway that is involved in both inflammation and angiogenesis. 

## 5. Conclusion

In this study, we demonstrated that *γ*-PGA significantly attenuated the symptoms and histological features of IBD, and reduced inflammation in an animal model of colitis; moreover, *γ*-PGA significantly reduced VEGF-A and VEGFR2 expression, indicating that the mechanism of action may involve a reduction in pathological angiogenesis. Taken together, our results suggest that *γ*-PGA has potential application in conditions marked by inflammatory-driven angiogenesis and mucosal inflammation. Therefore, *γ*-PGA may be a useful antiangiogenic/anti-inflammatory agent.
